# Cross-Sectional Surveys of the Prevalence of Follicular Trachoma and Trichiasis in The Gambia: Has Elimination Been Reached?

**DOI:** 10.1371/journal.pntd.0004906

**Published:** 2016-09-19

**Authors:** Sarah E. Burr, Ansumana Sillah, Anselme S. Sanou, Anita C. Wadagni, John Hart, Emma M. Harding-Esch, Sarjo Kanyi, Robin L. Bailey

**Affiliations:** 1 Department of Clinical Research, London School of Hygiene and Tropical Medicine, London, United Kingdom; 2 Disease Control and Elimination Theme, Medical Research Council Unit, Fajara, The Gambia; 3 National Eye Health Programme, Ministry of Health and Social Welfare, Kanifing, The Gambia; 4 West Africa Health Organization, Bobo-Dioulasso, Burkina Faso; 5 HIV/STI Department, Public Health England, London, United Kingdom; RTI International, UNITED REPUBLIC OF TANZANIA

## Abstract

**Background:**

The Gambia’s National Eye Health Programme has made a concerted effort to reduce the prevalence of trachoma. The present study had two objectives. The first was to conduct surveillance following mass drug administrations to determine whether The Gambia has reached the World Health Organization’s (WHO) criteria for trachoma elimination, namely a prevalence of trachomatous inflammation—follicular (TF) of less than 5% in children aged 1 to 9 years. The second was to determine the prevalence of trichiasis (TT) cases unknown to the programme and evaluate whether these meet the WHO criteria of less than 0.1% in the total population.

**Methodology/Principal Findings:**

Three cross-sectional surveys were conducted between 2011 and 2013 to determine the prevalence of TF and TT in each of nine surveillance zones. Each zone was of similar size, with a population of 60,000 to 90,000, once urban settlements were excluded. Trachoma grading was carried out according to the WHO’s simplified trachoma grading system. The prevalence of TF in children aged 1 to 9 years was less than 5% in each surveillance zone at each of the three surveys. The prevalence of TT cases varied by zone from 0 to 1.7% of adults greater than 14 years while the prevalence of TT cases unknown to the country’s National Eye Health Programme was estimated at 0.15% total population.

**Conclusions/Significance:**

The Gambia has reached the elimination threshold for TF in children. Further work is needed to bring the number of unknown TT cases below the elimination threshold.

## Author Summary

Trachoma, the world’s leading infectious cause of blindness, is caused by ocular infection with the bacterium *Chlamydia trachomatis*. The Gambia, situated in West Africa, has implemented all facets of the World Health Organization-recommended SAFE strategy for trachoma control including surgery to correct the in-turning of eyelashes (trichiasis), mass drug administration with antibiotics, promotion of facial hygiene and environmental improvements. In 2011, The Gambia’s National Eye Health Programme began three years of rolling surveys to determine the prevalence of trachoma in the country and to evaluate whether trachoma elimination has been reached. The results suggest the country has reached the elimination threshold for trachoma in children (less than 5% prevalence) but that more work needs to be done to reduce the prevalence of trichiasis in adults.

## Introduction

Trachoma, caused by the obligate intracellular bacterium *Chlamydia trachomatis*, is believed to be endemic in at least fifty countries, primarily in Africa and Asia [1]. The disease is characterized by repeated episodes of follicular conjunctivitis in childhood, resulting in progressive scarring of the upper eyelid, in-turning of the eyelashes (trichiasis; TT) and corneal opacification in later life [2]. Recognising that concerted action to control the disease was needed, the World Health Organization (WHO), in 1996, proposed the ‘SAFE’ strategy of trachoma control: Surgery to correct trichiasis, **A**ntibiotics to treat ocular chlamydial infection, and the promotion of **F**acial cleanliness and **E**nvironmental Improvement to address risk factors associated with the transmission of the infection [3].

The Gambia, situated in the Sahel of West Africa, has done much to implement the SAFE strategy to control trachoma within its borders. In 1986, following a national survey that determined trachoma was the second leading cause of blindness in the country [4], the National Eye Health Programme (NEHP) was formed and a network of community ophthalmic nurses was trained to screen communities for active trachoma and to conduct trichiasis surgery. Public health initiatives with a focus on preventative eye care and facial cleanliness have been targeted to school children and rural communities while urban centres have benefitted from a targeted programme designed to meet the eye health needs of marginalised populations. In 2007, a mass drug administration (MDA) programme, which distributed more than 400,000 doses of azithromycin, was rolled out in twenty-three priority health districts. Most recently, trichiasis case-hunting and surgery camps have been carried out across the rural regions of the country.

In 1996, a second survey of blindness found the prevalence of active trachoma in children aged 0 to 9 was approximately 7% nationally [5]. A decade later, population-based surveys conducted in two regions in advance of the MDA campaign found greater than 10% prevalence of trachomatous inflammation—follicular (TF) in the same age group [6]. Based on these data, eleven rural districts with predicted prevalence greater than 10% were earmarked for three rounds of MDA with azithromycin according to the WHO criteria. Treatment began in 2007 and finished in 2010. In another twelve districts where the prevalence of TF in children was projected to be between 5 and 10%, a screen and treat strategy was adopted. Under this scheme, community-based screening of all children aged 1 to 9 years was first carried out. If the prevalence of TF in 1 to 9 year olds was greater than 10%, the community was selected for three rounds of MDA. If the prevalence was less than 10%, households of active cases were treated with one round of MDA. In 2011, the Partnership for the Rapid Elimination of Trachoma, or PRET study, surveyed four formerly endemic districts by conducting screening in villages that had received either one round of MDA three years previously or three rounds of MDA completed one year previously. The results demonstrated very low levels of TF and ocular chlamydial infection in all communities surveyed and suggested the country might have reached the elimination benchmark of TF prevalence less than 5% in children [7].

The WHO has convened global scientific meetings on post-endemic surveillance for trachoma and these have established criteria for the elimination of trachoma as a public health problem [8, 9]. These criteria indicate that, following three years of surveillance, countries would be required to demonstrate, through appropriately powered surveys of rural areas, that (i) the prevalence of TF among 1–9 year olds was less than 5% and (ii) that the prevalence of ‘unknown’ cases of trichiasis (those that have never been offered surgery) was less than one per thousand (0.1%) of the total population in each district. A district was defined notionally as a unit of 250,000 population. With this advice in mind, the NEHP conducted a rolling programme of rural trachoma surveys over three years designed to show that TF prevalence remained low and that elimination criteria had been met. TT prevalence was also assessed and the results generated were used to calculate the number of TT cases unknown to the NEHP throughout the country.

## Methods

### Study site

**Fig 1:**
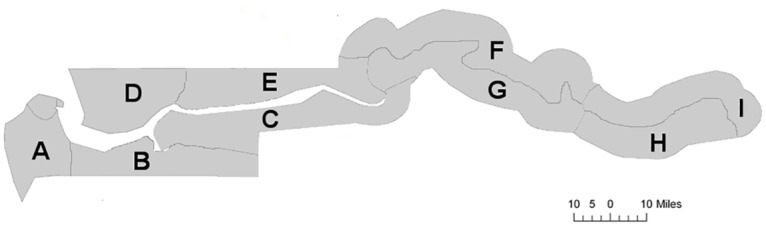
Map of The Gambia indicating the boundaries of the trachoma surveillance zones.

The regions of The Gambia were divided, on the basis of their population in the 2003 national census, into nine zones (A-I; Fig 1). Gambian administrative districts are significantly smaller than those in many other countries and the zones were constructed to be functionally equivalent to ‘sub-districts’ by amalgamating multiple contiguous districts to give similar sized units of 60,000–90,000 population. Urban settlements, as designated by The Gambia Statistics Department [10], were excluded as per the WHO recommendations.

### Sampling strategy

A sample size of 1,448 individuals for each population unit of 60,000 to 90,000 (zones A to I) was deemed necessary to estimate a prevalence of TF, in children aged 1 to 9 years, of 3% within a precision of +/- 2% given a 95% confidence limit and a design effect of 4. As sampling was intended to proceed over 3 years, this equated to 483 individuals aged 1 to 9 to be examined per zone, per year. The average Gambian household was estimated to contain 9 people, of whom 3.5 are in the age range 1–9; to achieve the required sample size, 138 households would need to be screened in each zone, each year. We therefore chose to conservatively target 160 households annually in each of the surveillance zones. This was achieved by selecting 16 settlements in each zone by probability proportional to size, conducting a census to list all households in each settlement and then screening ten randomly selected households from the list in each settlement for ocular examinations. A further two households were randomly selected as reserve households in the case of selected households being unavailable or choosing not to participate. In the case of large settlements (>1,000 population), segments of the village, defined on the basis of landmarks by the national census data, were selected for screening.

Based on the 2011 and 2012 results, the sample size was doubled in 2013 in order to have TF prevalence data in all zones from a single survey adequately powered to demonstrate that TF was less than 5% in each zone and thus fulfil the criteria for elimination.

### Data collection

Data collection took place from July 2011 to June 2013. Prior to the onset of field-work, a workshop was held for all community ophthalmic nurses and healthcare workers involved in the surveys. Training included quality control exercises of trachoma grading from slides in a classroom setting followed by practical exercises in the field. Graders were required to achieve agreement (kappa coefficients of 0.8 or more) for TF with a senior grader (RB) for the slide grading exercise, which consisted of a variety of images from The Gambia, Niger and Tanzania and which were used in the validation system for the PRET study [7]. During the surveys, all household members, defined as those who slept in the household the night before, were eligible for screening. Consenting individuals were examined for clinical signs of trachoma using a 2.5× magnifying loupe and adequate sun or torch-light. Trachoma grading was carried out according to the WHO simplified grading system [11]; TF was defined as the presence of five or more follicles, greater than 0.5mm diameter, on the upper tarsal conjunctiva and TT was defined as at least one lash touching the globe, or evidence of epilation.

### Data entry and analysis

Household data were summarised from paper survey forms as the number of children aged 1–9, the number of cases of TF in children aged 1–9, the number of individuals aged more than 14 and the number of TT cases in those over 14 in the household. These summary data were entered into a Microsoft Access database by NEHP staff and subsequently analyzed in Stata version 12.1 (StataCorp LP, College Station, USA). Confidence intervals were calculated using the Wilson score interval [12].

### Case management

All cases of TF along with their household contacts were treated with a single oral dose of azithromycin as is NEHP policy. Pregnant women and infants under the age of 6 months were instead offered tetracycline eye ointment. Individuals with TT were offered Trabut surgery free of charge.

### Ethical review

The study adhered to the tenants of the Declaration of Helsinki and was approved by The Gambia’s Ministry of Health and Social Welfare and by The Gambia Government/Medical Research Council Unit, The Gambia Joint Ethics Committee. Written, informed consent was obtained from all participants; in the case of minors, informed consent was obtained from the parent or guardian.

## Results

### Trachoma prevalence

Over the course of the study period, we examined 19,205 children aged 1 to 9 years and 27,921 adults over the age of 14 years for trachoma. The prevalence of TF in children varied by zone in each of the three years (Table 1), ranging from 0 to 3.8% [95% Confidence Interval (CI), 2.5–5.9]. In 2013, the final year of the survey, TF prevalence ranged from 0.2% (95% CI 0.1–0.9) to 3.2% (95% CI 2.3–4.4)

**Table 1:** Prevalence of TF in children aged 1 to 9 years by surveillance zone in years 2011 to 2013.

	2011		2012		2013	
Zone	n/N	% (95% CI)	n/N	% (95% CI)	n/N	% (95% CI)
A	5/402	1.2 (0.5–2.9)	15/441	3.4 (2.1–5.5)	10/833	1.2 (0.7–2.2)
B	8/460	1.7 (0.9–3.4)	11/605	1.8 (1.0–3.2)	18/953	1.9 (1.2–3.0)
C	0/444	0	5/427	1.2 (0.5–2.7)	13/712	1.8 (1.1–3.1)
D	7/498	1.4 (0.7–2.9)	19/496	3.8 (2.5–5.9)	37/1163	3.2 (2.3–4.4)
E	0/412	0	3/459	0.7 (0.2–1.9)	2/829	0.2 (0.1–0.9)
F	11/556	2 (1.1–3.5)	17/679	2.5 (1.6–4.0)	7/1111	0.6 (0.3–1.3)
G	3/548	0.5 (0.2–1.6)	0/360	0	21/1019	2.1 (1.4–3.1)
H	4/763	0.5 (0.2–1.3)	7/1043	0.7 (0.3–1.4)	5/1111	0.5 (0.2–1.0)
I	1/602	0.2 (0.03–0.9)	20/629	3.2 (2.1–4.9)	6/1650	0.4 (0.2–0.8)

CI = Confidence Interval

Prevalence of TT varied by year and by zone from 0 to 1.7% (95% 1.0–3.0) (Table 2). In the final year of surveillance, TT prevalence ranged, by zone, from 0 to 0.6% (95% CI, 0.3–1.1).

**Table 2:** Prevalence of TT in adults greater than 14 years of age by surveillance zone in years 2011 to 2013.

Zone	2011		2012		2013	
	n/N	% (95% CI)	n/N	% (95% CI)	n/N	% (95% CI)
A	6/603	1 (0.5–2.2)	3/649	0.5 (0.2–1.4)	3/1140	0.3 (0.1–0.8)
B	2/694	0.3 (0.1–1.0)	8/662	1.2 (0.6–2.4)	8/1425	0.6 (0.3–1.1)
C	3/664	0.5 (0.2–1.3)	5/663	0.8 (0.3–1.8)	10/964	1 (0.6–1.9)
D	12/686	1.7 (1.0–3.0)	3/699	0.4 (0.1–1.3)	2/1362	0.1 (0.04–0.5)
E	2/590	0.3 (0.1–1.2)	5/665	0.8 (0.3–1.7)	4/1329	0.3 (0.1–0.8)
F	2/837	0.2 (0.1–0.9)	5/993	0.5 (0.2–1.2)	7/1561	0.4 (0.2–0.9)
G	0/900	0	0/774	0	2/1982	0.1 (0.03–0.4)
H	0/771	0	0/805	0	0/1514	0
I	0/1043	0	1/1152	0.1 (0.02–0.5)	3/2794	0.1 (0.04–0.3)

CI = Confidence Interval

### Burden of trichiasis

The projected number of trichiasis cases in each zone was estimated by first calculating the average TT prevalence in individuals aged over 14 and who were available to survey in each zone over the course of the three-year study. The average prevalence per zone was then multiplied by 60% of the rural population in each zone [60% being the estimated proportion of the Gambian population over 14 years of age and assuming that TT in young children is negligible (no cases were observed among 19,205 children examined in the survey)] to determine an approximate number of projected cases living in each zone (Table 3). Using this method, the total number of projected cases across each of the nine zones was estimated to be 1,594.

**Table 3:** Projected number of TT cases by surveillance zone.

Zone	Total rural population	Projected cases	Known cases^a^	Projected unknown cases	Prevalence unknown cases^b^ (%)
A	82,082	282	54	228	0.28
B	71,904	296	64	232	0.32
C	58,865	264	136	128	0.22
D	76,282	355	71	284	0.37
E	61,370	171	133	38	0.06
F	73,451	175	58	135^c^	0.08^c^
G	87,150	18			
H	74,420	0	54	0^d^	0^d^
I	84,508	33			
Total	670,032	1594	570	-	-

^a^ Cases documented at dedicated eye health clinics

^b^ Prevalence unknown cases per total population

^c^ Zones F and G served by one dedicated eye health clinic

^d^ Zones H and I served by one dedicated eye health clinic

In an effort to determine the number of trichiasis cases known to the NEHP, the trichiasis registries held at seven of the eight major health facilities with dedicated eye health units were examined. One registry was unavailable. 570 TT cases were recorded in these registries. We therefore estimate the total number of ‘unknown’ cases to be 1,024 (1,594 minus 570), which represents 0.15% of the total rural population.

## Discussion

The criteria for trachoma elimination, as outlined by the WHO, include less than 5% prevalence of TF in children aged 1–9 years and less than 0.1% prevalence unknown TT in the total population [9]. Our results indicate that The Gambia has reached TF elimination in all surveillance zones. The measured TF prevalence and the upper 95% confidence bound were under 5% in all zones at all time points with the exception of two zones in 2012, where the upper confidence bounds were 5.5 and 5.0%. In 2013 however, these values were lower.

Re-introduction of ocular *C*. *trachomatis* infection after mass treatment has been documented in The Gambia, primarily through interaction with untreated communities in Senegal [7, 13]. We also have recent antedotal evidence of migrants, some with active disease, leaving Senegal and resettling in Gambian communities, potentially providing a means of re-infection. However, it is not yet clear whether isolated re-infection events are enough to re-establish transmission following local elimination [7]. Continued TF surveillance will be important for The Gambia and preliminary studies to evaluate the utility of serological tools to identify future transmission events [14] have recently been conducted in the country.

We have estimated the number of ‘unknown’ trichiaisis cases in The Gambia at 1,024. This represents roughly 0.15% of the total rural population or 1.5 cases per thousand and suggests the elimination criteria for TT may not yet have been met. While the projected prevalence of unknown TT cases in Zones E through I are in-line with the elimination threshold, increased numbers in Zones A to D, representing the western-most part regions of the country are above the target currently set for elimination. Further resources directed toward active case hunting may be needed to identify those TT cases that are not yet known to the NEHP.

The assessment of TT has received attention with the recognition that many countries, including those that have made progress in reducing TF through trachoma control measures, have substantial TT surgery backlogs [15, 16]. The rationale for the elimination threshold as cases ‘unknown’ to the programme is that this best reflects this surgical backlog however, we found this number difficult to reconstruct from the available programme records. Dedicated TT registries were found in all the eye health units we visited. All registers were hand-written in bound notebooks. In several of the units, TT cases diagnosed in the health centre were recorded in notebooks separate from those cases identified during community-screening exercises, which made the total number of TT cases identified in a given year more cumbersome to determine. A third registry was often then used to record TT surgeries conducted. While a number of units made a note of those individuals who were diagnosed with TT but who then refused surgery, many did not. Because of this and because different notebooks were used to identify cases and record surgery details, there was no obvious way of determining whether all cases had been offered appropriate treatment. Furthermore, the NEHP has continued to recommend epilation for minor TT (defined as 5 or fewer lashes rubbing the eyeball) and it is unclear how many of those classified as TT, on the basis of epilation, were epilating on the advice of the programme and therefore ‘known’ to the NEHP.

The Gambia is also lacking a robust means of documenting recurrent TT cases. Recurrent cases are entered into the NEHP’s trichiasis registries along with all other TT cases identified. While this means that recurrent TT is accounted for within the ‘known’ cases, it doesn’t enable one to calculate the total percentage of TT cases that are recurrent. This is important as elimination criteria indicate that countries must report recurrence rates with a target of achieving 10% or less recurrence one year after surgery [9]. The Gambia would benefit from a central, electronic TT registry that would enable data collection from across the country in a unified manner and to ensure appropriate follow-up care of all cases identified. The use of electronic data collection in the field has been shown to be feasible within the Global Trachoma Mapping Project, which uses Android smartphones that transmit data to a high security server for cleaning and approval by the relevant Ministry of Health [17]. A similar system could conceivably be developed to allow eye care units to report TT cases directly to the NEHP.

TT is diagnosed here according to the simplified classification of trachoma [11], this being at least one lash in contact with the eyeball, or evidence of epilation. It is questionable how accurately rural household surveys (of the kind needed to determine prevalence of TF) accurately identify TT surgical need. Firstly, it is clear that cases of trichiasis can be found in urban areas; a recent study in The Gambia found cases unknown to the programme within 200 metres of a functioning eye health unit [18]. Secondly, a survey based on households can only find cases of TT that are present in the household and certain age groups are typically under-represented in Gambian household surveys. This can be dealt with by standardising the estimates against a rural population pyramid, but it is unclear that this would correct the estimates appropriately if, for example, people with TT were more likely to be found at home during trachoma surveys. A final issue concerns the relationships between TT as diagnosed here, trachoma and surgical need. The surgical procedures for TT, typically bilamellar tarsal rotation or Trabut, are designed to correct entropion, the in-rolling of the lid margin that characteristically follows contraction of trachomatous scarring of the eyelid [19]. However, not all trichiasis is associated with entropion; neither lashes rubbing the eyeball nor epilation need be trachomatous in origin [20]. What may be needed is a methodology for TT surveys that are designed to better identify surgical backlog and that require TT be designated as trachomatous only when trachomatous scarring is also present.

Our data, although limited for the reasons outlined above, estimate that rural Gambia has a backlog of around 1,000 TT cases requiring surgery, against the elimination threshold allowance of 1 per thousand of the population, or approximately 670 cases. The difference of 330 is a relatively small number for a country where community ophthalmic nurses are trained to conduct Trabut surgery in health centres and in people’s homes and who, in the past three years, have conducted roughly 200 surgeries a year. With additional funding for enhanced case-finding and TT surgery camps, it should be possible to reduce this number with relative ease.

Virtually no TT was found in the most-eastern part of the country, encompassing the Upper River Region. This is consistent with historical data of TF prevalence in this region. The National Survey of Blindness carried out in 1986 documented a prevalence of active trachoma [TF and/or trachomatous inflammation—intense (TI)] in children aged 0 to 14 years of 5% in Upper River Region, half that of the prevalence found in the country as a whole [4]. By 1996, this value had further dropped to 1.2% prevalence [5]. As the prevalence of TF in the rest of the country has now reached these low levels, one may speculate that TT has the potential to disappear from Gambian communities within the next two generations.

The estimates of TT cases remaining in the country are based on national census data from 2003, which is a further limitation of the study. The Gambia Statistics Department conducted another nation-wide census in 2013 but the complete data-set from this survey has yet to be released. However, the preliminary data that have been made public [21] suggest the bulk of the population increase over the past decade has occurred in the urban areas, which are excluded by the WHO guidelines for trachoma surveillance. We therefore do not anticipate the projected numbers of TT cases will change dramatically once re-evaluated based on the 2013 census data.

### Conclusions

Data collected during three years of rolling surveys indicate The Gambia has achieved the elimination criteria for TF in children, representing a significant public health achievement. Further efforts in identifying and a more robust system of documenting TT cases would help reduce the number of unknown TT cases from the current figure of 0.15% total population to below the 0.1% population elimination threshold.

## Supporting Information

S1 Checklist STROBE checklist. (DOC)
